# Checkpoint inhibitor induced cardiotoxicity: managing the drawbacks of our newest agents against cancer

**DOI:** 10.18632/oncotarget.22579

**Published:** 2017-11-21

**Authors:** Karina Brüstle, Bettina Heidecker

**Affiliations:** Bettina Heidecker: Department of Cardiology, University Hospital Zurich, Zurich, Switzerland

**Keywords:** immune therapy, checkpoint inhibitors, myocarditis

Immune therapy has shifted the paradigm of modern cancer treatment: Instead of targeting the tumor itself, it focuses on enhancing tumor recognition and destruction. Harnessing the immune system by using monoclonal antibodies, adoptive T cell transfer or checkpoint inhibitor (CPI) therapy increased progression free survival with less side effects compared to conventional therapy [1]. Checkpoint inhibitors focus on „double inhibition“ and therefore activation of T cells. IgG Antibodies against CTLA4 (Ipilimumab), PD-1 inhibitors (Nivolumab, Pembrolizumab) and PD-L1 inhibitors (Atezolizumab) have been approved as first line therapy for malignancies such as metastatic melanoma and non-small cell lung. However, the benefits of an activated immune system come with a price - the risk of immune related adverse events such as myositis, cardiovascular complications, skin rash, colitis, hepatitis, or endocrinopathies. Cardiotoxicity including arrhythmias, conduction defects, heart failure, and fulminant myocarditis with fatal outcome have been reported since establishing CPI therapy [2]. An autopsy study revealed that not all immunologic effects of CPIs on the heart become clinically apparent, despite some level of myocarditis with a CD8+ T cell predominant lymphocytic infiltrate on histology. Murine studies demonstrate that cardial PD-1 protects the heart against T cell mediated inflammation and that mice, upon PD-1 inhibition, develop myocarditis [3]. Furthermore, sequencing of the T cell receptor CD3 shows shared sequences in tumor, cardiac and skeletal muscle, suggesting fatal myocarditis to result from common antigen response [4]. Studies have shown that combination of two immunotherapeutic agents elicited more immune related side effects vs monotherapy.

Three of the major questions that arise for caregivers and scientists are: 1) Should pre-therapeutic screening be implemented to assess risk for fatal cardiovascular events and adjust mono- vs. combination CPI therapy? Certain features of the immune system may predispose to immune related adverse events, such as specific HLA phenotypes or composition of their T cell subtypes. 2) Should patients undergoing CPI therapy receive regular surveillance tests for early signs of cardiovascular adverse events? Which non-invasive technologies should be used? 3) Would local therapy be beneficial if cardiotoxicity develops? During CPI therapy, troponin elevation, changes in ECG, arrhythmias, and inflammation or ischemia on cardiac MRI have been observed -clinical signs that will likely guide therapy in the future. In some reported cases that tested for the most common viruses known to cause myocarditis (cocksackievirus, parvovirus B19, human herpesvirus 6 and other adeno-/enteroviridae), viral serologies from peripheral blood were negative in all patients [2]. Myocardial biopsies typically show lymphocytic infiltrates with predominance of cytotoxic CD8 T cells and decreased FoxP3+ regulatory Tcells. While endomyocardial biopsy remains the gold standard for the definitive diagnosis of myocarditis, its use is recommended in: 1) Heart failure requiring inotropic or mechanical circulatory support, 2) Mobitz type 2 second degree or higher heart block, 3) sustained or symptomatic ventricular tachycardia or 4) failure to respond to guideline based medical management within 1-2 weeks (class of recommendation I, level of evidence B) . Cardiac MRI may is a valuable non-invasive alternative, if myocarditis is suspected [5]. For general surveillance during CPI treatment, we recommend a physical exam, routine laboratory parameters including troponin, and ECG in regular intervals (every 1-2 months). An echocardiogram at baseline should be obtained and repeated if there is a change in clinical status suggesting compromised cardiovascular function. In that scenario, a cardiac MRI may be considered as well.

**Figure 1:**
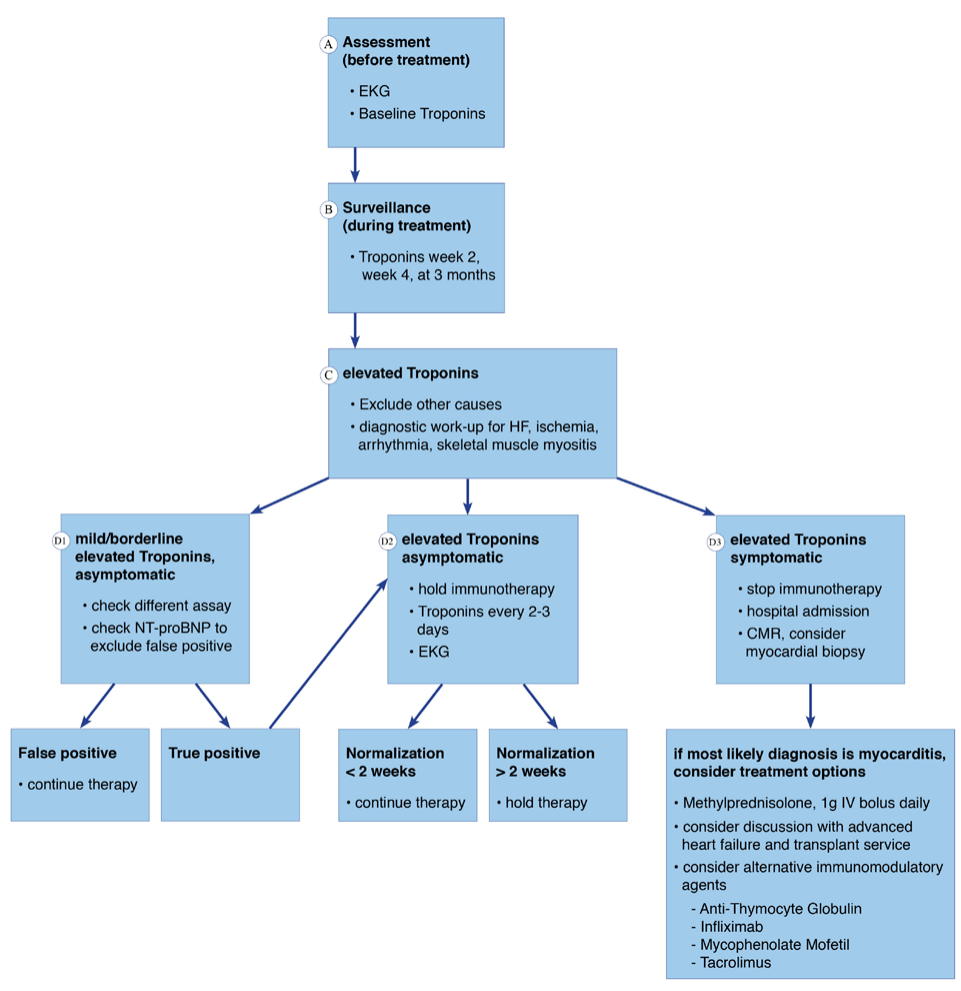
Management of immune-mediated myocarditis

In the event of increasing troponin levels, Wang and colleagues recommend to hold immunotherapy until troponin levels and ECG abnormalities normalize (Figure 1) [6]. In a symptomatic patient with myocarditis, immunotherapy has to be stopped with subsequent initiation of high dose steroid treatment. In that scenario, endomyocardial biopsy may be considered to obtain a definitive diagnosis of myocarditis.

While these conclusions and recommendations reflect our interpretation of findings from case reports and case series, no guidelines for the treatment of immune related myocarditis have been published as of today due to limited availability of clinical data.

Assuming an immune antigen reaction similar to an allograft rejection or a rheumatic disease, similar pathomechanisms have to be explored to widen treatment options for this rare but often fatal complication. In one reported case of CPI related and steroid resistant myocarditis, treatment with equine anti-thymocyte globulin (ATGAM) led to regression of the disease [7]. Similar to cases of allograft dysfunction, additional immunosuppressive therapy with mycophenolate mofetil or calcineurin inhibitors may be beneficial. The histopathological findings of increased effector T cells and decreased regulatory T cells in patients with immune related adverse events suggests an imbalance of inflammatory and anti-inflammatory T cell subsets. This mimics the setting of chronic graft dysfunction in which upregulation of regulatory T cells to restore balance has been shown to decrease inflammation and reduce tissue damage [8]. Similar to auto-immune myocarditis in sarcoidosis, vasculitic disease, or lupus anti-rheumatic agents such as TNFalpha inhibitors or cyclophosphamide may be considered. Studies in animal models are necessary to elucidate the immunologic pathways and cross reactivities causing these adverse events. Meanwhile, caregivers should be aware of the classic risk factors for cardiotoxicity under CPI treatment: Hypertension, decreased left ventricular function, rhythm abnormalities, an predisposition to autoimmune disease. In challenging clinical scenarios with presence of several of these risk factors, an interdisciplinary approach as well as sharing of data of incidence and outcome with the scientific community is invaluable to make future immune therapy both safer and more efficient.
