# Correction: Checkpoint inhibitor induced cardiotoxicity: managing the drawbacks of our newest agents against cancer

**DOI:** 10.18632/oncotarget.26113

**Published:** 2018-09-07

**Authors:** Karina Brüstle, Bettina Heidecker

**Affiliations:** Department of Cardiology, University, Hospital Zurich, Zurich, Switzerland

**This article has been corrected:** The correct reference 5 is given below:

5. Berg JK, et al. Circulation Heart Failure. 2017; 10.

Original article: Oncotarget. 2017; 8:106165-106166. https://doi.org/10.18632/oncotarget.22579

